# Relative Importance of Sex, Pre-Starvation Body Mass and Structural Body Size in the Determination of Exceptional Starvation Resistance of *Anchomenus dorsalis* (Coleoptera: Carabidae)

**DOI:** 10.1371/journal.pone.0151459

**Published:** 2016-03-15

**Authors:** Michal Knapp

**Affiliations:** Department of Ecology, Faculty of Environmental Sciences, Czech University of Life Sciences Prague, Kamýcká 129, Praha 6 – Suchdol, CZ-165 21, Czech Republic; CNRS, FRANCE

## Abstract

In nature, almost all animals have to cope with periods of food shortage during their lifetimes. Starvation risks are especially high for carnivorous predatory species, which often experience long intervals between stochastic prey capturing events. A laboratory experiment using the common predatory carabid beetle *Anchomenus dorsalis* revealed an exceptional level of starvation resistance in this species: males survived up to 137 days and females up to 218 days without food at 20°C. Individual starvation resistance was strongly positively affected by pre-starvation body mass but only slightly by beetle structural body size per se. Females outperformed males even when the effect of gender was corrected for the effects of structural body size and pre-starvation body mass. The better performance of females compared to males and of beetles with higher relative pre-starvation body mass could be linked to higher fat content and lean dry mass before starvation, followed by a greater decrease in both during starvation. There was also a difference between the sexes in the extent of body mass changes both during ad libitum feeding and following starvation; the body masses of females fluctuated more compared to males. This study stresses the need to distinguish between body mass and structural body size when investigating the ecological and evolutionary consequences of body size. Investigation of the net effects of body size and sex is necessary to disentangle the causes of differences in individual performances in studies of species with significant sexual size dimorphism.

## Introduction

Under natural conditions, the majority of animals suffer from food shortages during some part of their life cycle [1,2]. The risk of starvation is especially high in animals utilizing food sources with unpredictable availability in time and space, as is frequent in predatory species. Carnivorous carabid beetles actively hunt for arthropods of various sizes, often of the same or even larger sizes than these predators, but long intervals can occur between successful prey capture events [3–9]. Thus, it is not surprising that adult carabids live under almost permanent food shortage conditions [10,11]. Starvation has substantial effects on fitness-related traits such as the well-known reduction in fecundity that occurs under a restricted food supply [12,13]. Starvation lasting long enough can result in death, an outcome that is not exceptional in nature. Among different taxa, longevity without food varies widely, but in general, ectotherms are able to survive longer starvation periods than endotherms [1]. However, current knowledge of the starvation resistance of carabid beetles is quite limited.

Many animal species also display substantial intraspecific variation in starvation resistance, the causes of which have been subjected to intensive research (e.g., [14–17]). At intra-population levels, body size and gender seem to be the most relevant determinants of individual starvation resistance [14,18–20]. In general, larger individuals outperform smaller ones, and females outperform males [14,18,19,21]. Unfortunately, studies simultaneously investigating the effects of both these crucial factors on starvation resistance are nearly absent in insects (but see [22]; and see [23] for a study investigating the effects of body size and gender on resistance to hydric stress). Moreover, the situation is complicated by the existence of sexual size dimorphism in the majority of insect species [24,25]. When females are systematically larger than males, we cannot be sure whether their higher starvation resistance is caused by their larger body sizes or by some other sex-specific physiological or behavioural traits. To resolve this puzzle, an analysis (for example, a variance partitioning analysis) of the relative importance of body size and gender is needed.

To discover the mechanism responsible for the higher or lower starvation resistance of particular individuals, more detailed measurements are necessary (e.g., quantification of energy stores and their consumption rates). For example, the storage and consumption rate of lipid content (mass) could differ between sexes or may scale with body size [22,26] (for intraspecific comparison see [27]). Typically, not all body parts grow equally as total body size increases (i.e., growth isometry), but allometric scaling is quite common [28]. A relative increase or decrease of energy reserves as body size increases could be a good indicator of starvation resistance that increases or decreases depending on the total body size of particular species. The energy needed for maintenance of the tissues necessary for insect life could be covered by sugar and lipid storage as well as by proteins via tissue resorption [9,14,29,30]. Sex-specific starvation resistance could be not only a result of differences in the content of particular tissues/compounds that enable energy acquisition but also could be the result of sex-specific abilities to utilize particular tissues/compounds for energy acquisition [14].

This study investigated the relative importance of structural body size, sex and pre-starvation body mass in determination of starvation resistance in the carabid beetle *Anchomenus dorsalis*. To discover the mechanism underlying the observed starvation resistance pattern, the lipid content and lean dry mass of fully fed and completely starved males and females were measured, and linkages between structural body size, sex and pre-starvation body mass and lipid content and lean dry mass were investigated. A subset of investigated specimens was also measured for temporal changes in live mass in response to ad libitum feeding and to subsequent starvation until death.

## Materials and Methods

### Study species and specimen collecting

**Fig 1:**
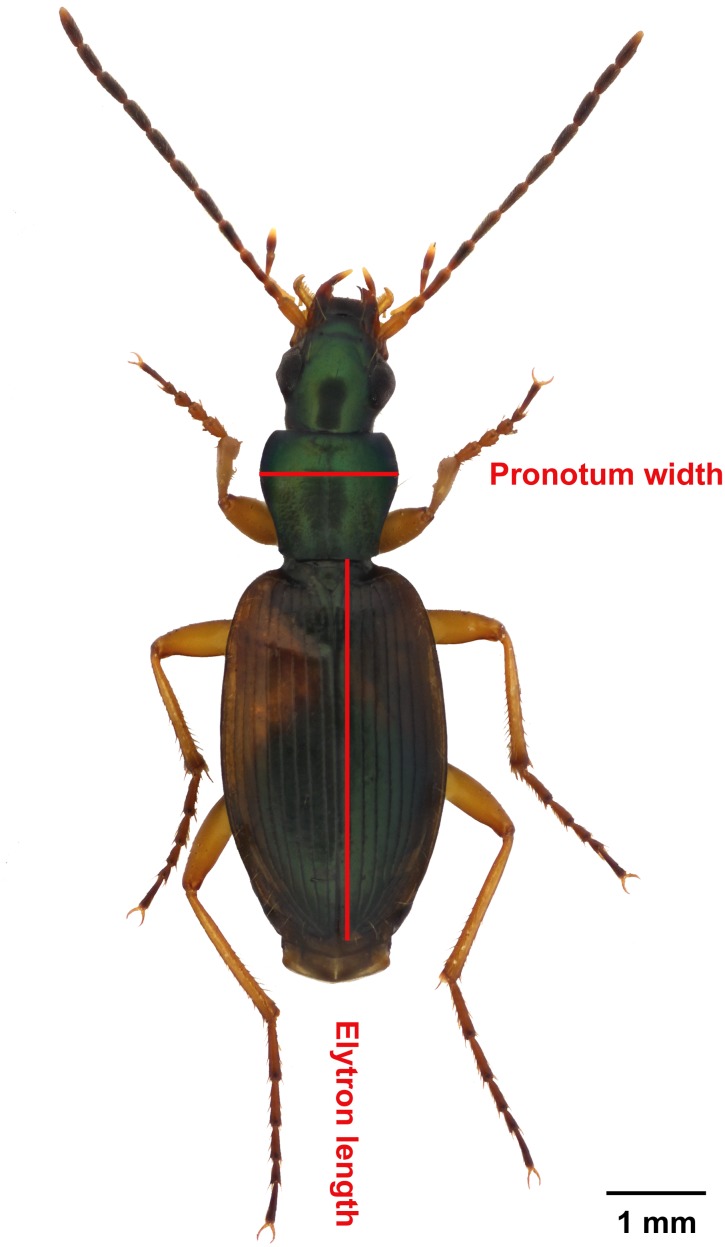
Carabid beetle *Anchomenus dorsalis* (Pontoppidan, 1763). Red lines indicate the measured dimensions of structural body size (elytron length and pronotum width).

*Anchomenus dorsalis* (Pontoppidan, 1763) (Coleoptera: Carabidae) is a medium-sized (5.6–7.7 mm long; Fig 1) open habitat generalist species [31]. This ground beetle reproduces in spring and early summer, similar to the majority of Central European carabid species. Its larval growth takes place during summer and adult beetles emerge in late summer and begin feeding to increase their energy reserves before overwintering [32]. Sexual size dimorphism and substantial variations in structural body size within each gender have been well documented for this species [33,34]. In the data of this study, the mean elytron length of females, 4.48 mm, was approximately 6% longer than that of males, 4.23 mm. *A*. *dorsalis* is a polyphagous predator that hunts for diverse arthropods, including those considered as serious pests, and should therefore be classified as a beneficial organism [5].

The insects employed in this study were individually collected using aspirators at the margins of arable fields near Prague-Suchdol, Czech Republic (GPS: 50°7'52"N, 14°21'49"E). Landowners agreed to the collecting activities, and no other specific permissions were required for collecting A. *dorsalis* because this common species is not protected by Czech law. Beetles were sampled in the second half of March 2011 immediately after they had finished overwintering in the grassy boundaries of neighbouring arable fields. In total, more than 400 individuals were collected and transported to the laboratory located in the Crop Research Institute (Prague-Ruzyně, Czech Republic) for subsequent investigation.

### Laboratory procedures

In the laboratory, beetles were sexed; subsequently, sexes were maintained separately to preclude mating. Altogether, 125 individuals of each sex were selected for subsequent experiments. To maximize body size variation among the experimental beetles, the 40 smallest and 40 largest individuals were selected (assessed visually) and an additional 45 medium sized individuals were selected at random for both sexes. Of these, 100 males and 100 females of various sizes, ranging from small to large, were selected for group rearing and randomly divided into groups of 20 specimens (5 groups per sex). Each group was placed into a large petri dish, 25 cm in diameter, with a sandy substrate. The remaining 25 males and 25 females (also ranging in size from small to large) were weighed to determine their live body mass (post-overwintering body mass) and placed individually into small plastic pots, 7 cm in diameter, with a sandy substrate. The groups as well as the individually reared beetles were subsequently provided with food and water ad libitum for 14 days. Mealworm (*Tenebrio molitor* L.) larvae (cut into pieces) and dog biscuits were provided as food. Water was supplied in modified Eppendorf^®^ tubes with cotton wool stoppers. Food and water were renewed twice a week, and all remnants of old food were removed at the same time to preclude mould infection. Beetles were reared (fed and subsequently starved) under controlled laboratory conditions at a constant temperature of 20°C, with a photoperiod of 16 hours of light and 8 hours of darkness.

### Measurement of live body mass changes (detailed dataset)

The beetles housed individually in plastic pots were weighed to determine their live mass twice a week. After 14 days with the ad libitum food supply, the food was removed, and the starvation experiment was started. Beetles were subsequently reared without any food, but supplied with water ad libitum. Live body mass measurements for each individually housed beetle (25 males and 25 females) continued twice a week for the next 11 weeks and then the measurement frequency was reduced to once per week until the death of the last specimen. To measure starvation resistance, longevity without food (in days) was noted for each particular beetle. Dead beetles were stored at -20°C for subsequent measurements of structural body size. The measurements of the individually reared individuals resulted in a “detailed dataset” that finally contained data for 24 males and 25 females. Data for one male was omitted because this beetle died from a fungal infection within a few days after the start of the starvation experiment.

### Group rearing (fattened and starved datasets)

After 14 days of ad libitum feeding, the collectively reared beetles were assessed visually for body size, and the 34 largest and 34 smallest beetles of each sex were selected. These beetles, which had extreme body sizes, were subsequently divided into two groups, resulting in 17 small males + 17 large males + 17 small females + 17 large females per group. Both groups were augmented by 16 males and 16 females of medium size selected at random from the remaining individuals so that a full range of body sizes was covered within each group. Individuals from the first group were kept for 24 hours without any food but supplied with the water. A period of 24 hours without any food was applied to minimize the effects of ingested food on measurements of body composition. After this period, beetles were weighed to determine live body mass and then killed by freezing. Killed beetles were stored at -20°C for subsequent body composition analyses (fat content, lean dry mass) and structural body size. Measurements of these beetles resulted in a “fattened dataset” that included data for 50 males and 50 females.

Individuals from the second group of beetles were given food ad libitum for 14 days, weighed for pre-starvation body mass, and then placed individually into plastic pots with a sandy substrate. Thereafter the beetles were reared without any food but supplied with water ad libitum. Water in modified Eppendorf^®^ tubes was renewed twice per week during the first 11 weeks and once per week during the following weeks. At the same time, dates of death were recorded for any dead individuals. Dead beetles were weighed and stored at -20°C for subsequent body composition measurements. Freezing seems to be an optimal storage technique for such purposes [34]. The measurements of these beetles resulted in a “starved dataset” that included data for 49 males and 50 females (one male died quickly due to a fungal infection and was omitted from the study).

### Fat content, lean dry mass and body size measurements

To assess the body composition of the collectively reared individuals (“fattened” and “starved” datasets), frozen dead beetles were dried at 50°C for 48 hours and subsequently weighed for dry body mass. Then, the fat extraction was performed. Each beetle was individually placed in an Eppendorf^®^ tube (1.5 ml) and submerged in a 1:1 (vol/vol) mixture of diethyl ether and chloroform for 72 hours [35,36]. Next, the beetles were dried again for 48 hours and subsequently weighed to obtain their lean dry mass. Fat content was calculated as the difference between the dry mass and lean dry mass. All body mass measurements were performed using a Sartorius^®^ analytical balance to a precision of 10^−5^ g. For all investigated beetles (“fattened dataset”, “starved dataset” and “detailed dataset”) individual body size measurements consisting of elytron length and pronotum width were taken using digital callipers to a precision of 0.01 mm (Fig 1). Two disparate dimensions were measured at once to cope with possible violated structural isomorphism among the specimens [37]. Complete raw data are available in S1 Dataset.

### Statistical analyses

To integrate two structural size measures (elytron length and pronotum width) into one variable representing structural body size in the following analyses, principal component analysis (PCA) was employed, and the score of each particular individual on the first ordination axis was used as the measure of its structural body size. The PCA was performed using Canoco 5 software [38]. All univariate models were computed using R 2.15.1 [39]. The significance of each term in each particular generalized linear model was tested using F tests (see below).

#### Analyses of live body mass changes

To investigate the effects of structural body size, sex and post-overwintering body mass on absolute mass gained during 14 days of post-overwintering feeding (i.e., pre-starvation body mass minus post-overwintering body mass), generalized linear models (GLM) with a gamma distribution of errors and a logarithmic link function were applied to the “detailed dataset”. The particular terms were ordered following this logical structure: 1) structural body size, 2) sex (to verify its significance even when its effect is corrected for the effect of structural body size), 3) log_10_ transformed post-overwintering body mass (to verify body mass significance even when its effect is corrected for the effect of structural body size and sex), and 4) all possible interactions among structural body size, sex and post-overwintering body mass. To investigate the effects of structural body size, sex and post-overwintering body mass on relative body mass gain, a GLM with a gamma distribution of errors and an inverse link function was employed with the response variable calculated as body mass gain divided by pre-starvation body mass after feeding ad libitum.

To investigate the effects of structural body size, sex and longevity without food (in days) on body mass decrease during the starvation experiment (i.e., pre-starvation body mass minus starved body mass), a GLM with a gamma distribution of errors was applied to the “detailed dataset”. The particular terms were ordered as follows: 1) structural body size, 2) sex, 3) longevity without food, and 4) all possible interactions among structural body size, sex and longevity without food. To investigate the effects of structural body size, sex and longevity on relative body mass decrease during starvation, a GLM with a gamma distribution of errors and an inverse link function was employed with the response variable calculated as body mass loss during starvation divided by pre-starvation body mass.

#### Analyses of determinants of individual starvation resistance

To analyse the effects of structural body size, sex and pre-starvation body mass on longevity without food, a GLM with a quasi-Poisson distribution of errors was applied to the dataset comprising all starved beetles (pooled “detailed dataset” and “starved dataset”). A quasi-Poisson distribution of errors was employed because of substantial overdispersion. To compare the relative importance of structural body size, sex and pre-starvation body mass in the determination of starvation resistance, a variance partitioning technique was applied, i.e., GLMs with an altered order of main terms were performed (for the principles of the variance partitioning technique see [40]). To investigate the net effect of pre-starvation body mass corrected for sex and structural body size on starvation resistance, the terms were ordered as follows: 1) structural body size, 2) sex, 3) log_10_ transformed pre-starvation body mass, and 4) all possible interactions between structural body size, sex and pre-starvation body mass. In fact, body mass corrected for structural body size represents a variable called “body condition” in many ecological studies. To investigate the net effect of sex corrected for structural body size and pre-starvation body mass on starvation resistance, the terms were ordered as follows: 1) structural body size, 2) log_10_ transformed pre-starvation body mass, 3) sex, and 4) all possible interactions. Finally, to investigate the net effect of structural body size corrected for sex on starvation resistance, the terms were ordered as follows: 1) sex, 2) structural body size, 3) log_10_ transformed pre-starvation body mass, and 4) all possible interactions. Structural body size was not corrected for pre-starvation body mass because this setting would have led to investigation of body condition (as was made in the analysis of net effects of pre-starvation body mass). The more important question for this study is whether body size alone determines individual starvation resistance, in other words, whether beetles of a particular sex differing in structural body size also differ in their starvation resistance. A finding of missing difference in starvation resistance between beetles of various body sizes and a significant net effect of sex will indicate the existence of some sex-specific physiological or behavioural adaptations that increase starvation resistance in the more resistant sex. An alternative analysis of individual survival during starvation was performed using the Cox proportional hazards model (see S1 Table for model results) [41].

#### Analyses of starvation effects on fat content and lean dry mass

To analyse the effects of structural body size, sex, pre-starvation body mass, starvation treatment (beetles fattened or starved to death) and their interactions on fat content of experimental beetles, a GLM with a gamma distribution of errors was applied to the pooled dataset (“fattened dataset” and “starved dataset”). Investigated independent variables are correlated. Females have a systematically higher structural body size than males (t = 7.06, P < 0.001), and beetles with a larger structural body size have a systematically larger pre-starvation body mass (r = 0.84, P < 0.001). Therefore, the significance of the net effect of each particular variable was tested (i.e., other variables were employed as covariates in the model). Analogous models to these for absolute fat content were also applied to investigate the effects of structural body size, sex, pre-starvation body mass, starvation treatment and their interactions on absolute lean dry mass.

To investigate the net effects of structural body size, sex, pre-starvation body mass and their interactions on relative fat content (the portion of pre-starvation body mass assigned to lipids) of fattened beetles, GLMs with a gamma distribution of errors and an inverse link function were performed for the “fattened dataset”. Analogous models to these for relative fat content were also applied to investigate the effects of structural body size, sex, pre-starvation body mass and their interactions on relative lean dry mass (the portion of pre-starvation body mass assigned to lean dry mass).

## Results

### Changes in live body mass

**Fig 2:**
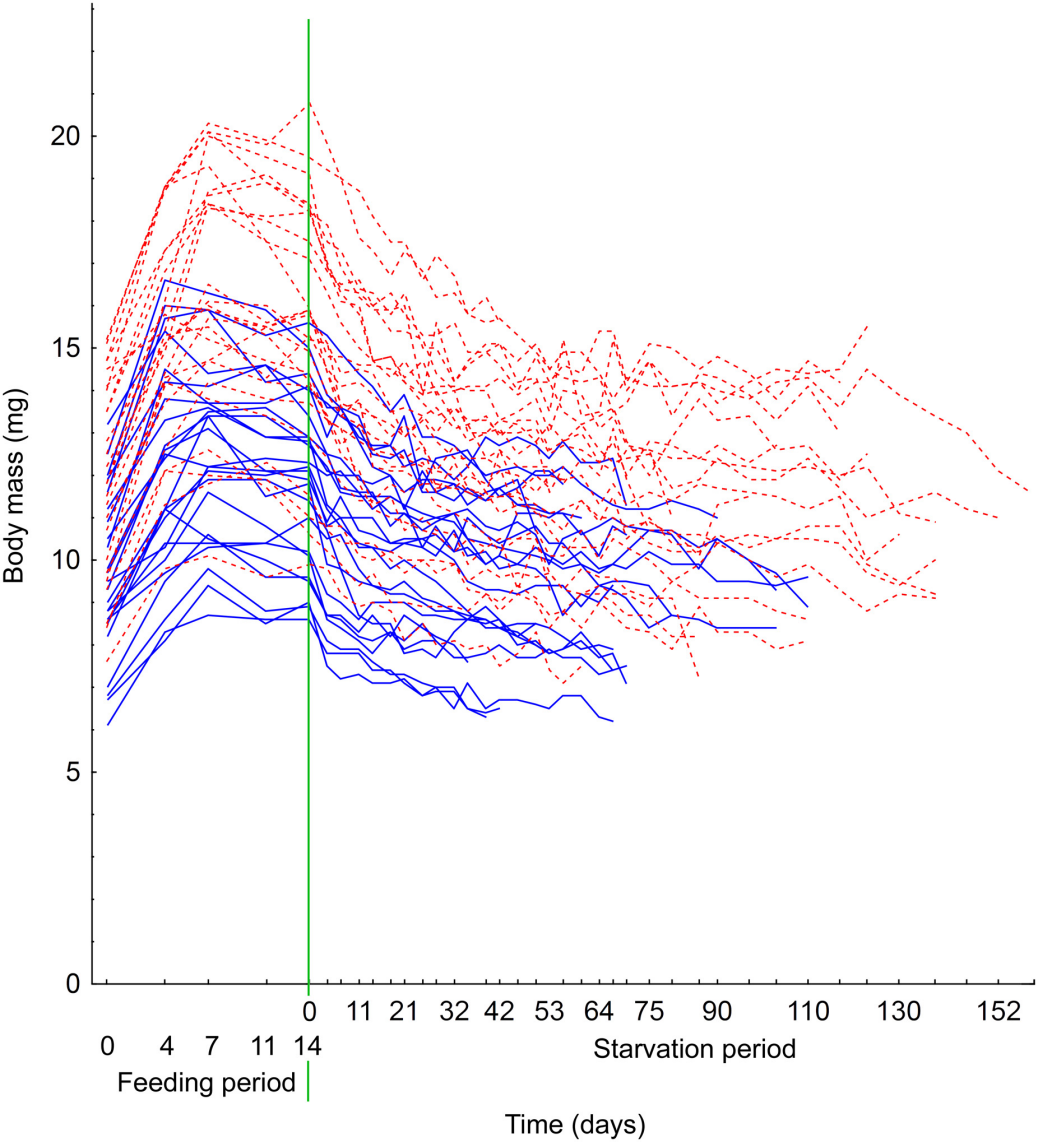
Live body mass changes in *Anchomenus dorsalis* during ad libitum feeding and starvation periods. The body mass of each individual beetle (25 females = red dashed lines; 24 males = blue lines) was measured during the initial feeding period (on left from green line) as well as during starvation following the feeding period (on right from green line) until its death. Note that the scaling differs between the feeding and starvation periods to make the fast body mass increase during the first 7 days of the feeding period more visible. Short-term increases in body mass during the starvation period could be caused by variations in water content.

Post-overwintering beetles were able to achieve their maximal body mass during the first seven days of intensive feeding. Allotting additional time for unlimited feeding resulted in constant or slightly decreased live body mass in *Anchomenus dorsalis* (Fig 2). The absolute gain in live body mass was significantly positively affected by structural body size; gain of body mass was higher in females than in males, and individuals with lower relative body mass before food provision put on more weight than those with higher relative starting mass. There were also significant interactions between body size and sex and between body size and post-overwintering body mass. Mass gain resulting from increasing size was steeper in females than in males and mass gain was highest for large beetles with relatively low post-overwintering body mass. The relative gain in body mass resulting from intensive feeding was significantly higher in females compared to males (average male mass increased by 26% and average female mass by 31%). Beetles with low post-overwintering body mass increased their mass relatively more than beetles with high post-overwintering mass and there was also a significant interaction between body size and sex. Small males increased their body mass relatively more than large ones, whereas there were no differences between variously sized females (Table 1).

**Table 1:** Factors affecting body mass changes in *Anchomenus dorsalis* during feeding ad libitum and starvation periods. Reported results are based on generalized linear models with a gamma distribution of errors and a logarithmic link function for absolute mass changes or an inverse link function for relative mass changes. Relative mass changes are related to pre-starvation body mass (after 14 days of ad libitum feeding) that represents a 100% value for each individual beetle. Only significant terms affecting body mass gain (during the 14-day feeding period after beetle overwintering) or body mass loss (during the starvation period following the feeding period) are listed. Significant effects are highlighted in **bold**. A non-significant main effect was included in the final models when significant interaction including this term is present.

Response	Term	Df	Deviance	F-value	P-value	Df	Deviance	F-value	P-value
Mass gained	Structural size	1	1.864	15.38	**<0.001**	1	0.031	0.34	0.562
	Sex	1	0.690	5.70	**0.021**	1	0.438	4.78	**0.034**
	Pre-starvation body mass	1	1.620	13.37	**<0.001**	1	1.745	19.06	**<0.001**
	Structural size * Sex	1	0.848	7.00	**0.011**	1	0.512	5.60	**0.022**
	Structural size * Pre-starvation body mass	1	0.573	4.72	**0.035**				
	Total explained deviance		5.595				2.726		
	Residual deviance	43	8.036			44	7.346		
Mass lost	Structural size	1	2.292	37.90	**<0.001**				
	Sex	1	0.490	8.10	**0.007**	1	0.249	6.41	**0.015**
	Longevity	1	0.286	4.74	**0.035**				
	Sex * Longevity	1	0.357	5.91	**0.019**				
	Total explained deviance		3.425				0.249		
	Residual deviance	44	2.630			47	2.096		

Calculation of temporal changes in relative daily mass losses indicates that the decrease in *A*. *dorsalis* live body mass caused by starvation (no food, but water was provided ad libitum) was steeper during the first 14 days of starvation (on average, 13.5% of pre-starvation body mass was lost during this period), followed by a period of less rapid body mass loss (Fig 2). The absolute decrease of live body mass during starvation was significantly positively affected by body size, and the decrease was larger in females compared to males. There was also a significant interaction between sex and longevity without food. The decrease in body mass was positively linked to longevity in males, whereas in females the body mass loss was nearly independent of their longevity without food. However, the relative body mass loss was significantly higher in females than in males (Table 1).

### Longevity of starved beetles

**Fig 3:**
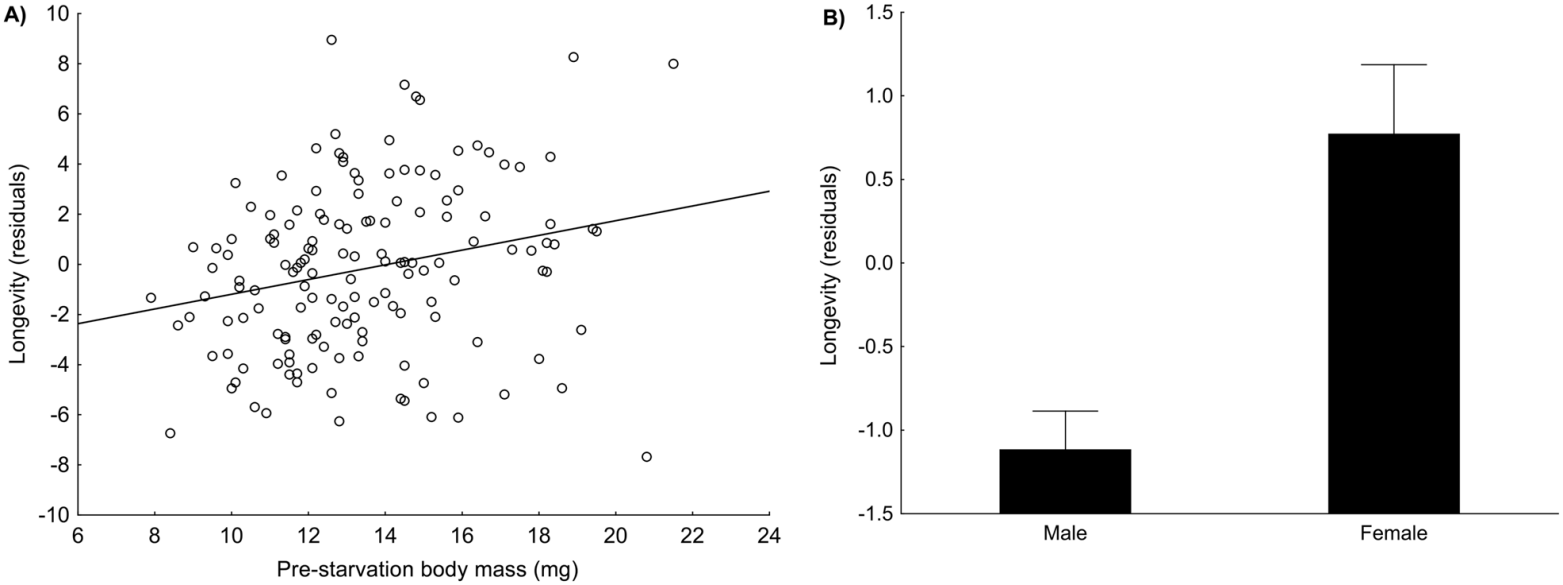
Net effects of pre-starvation body mass (i.e., body condition) and sex on starvation resistance. A) Relationship between pre-starvation body mass and longevity residuals (longevity without food corrected for effects of structural body size and sex). B) Relationship between sex and longevity residuals (longevity without food corrected for effects of structural body size and pre-starvation body mass). Means ± SE are shown.

**Fig 4:**
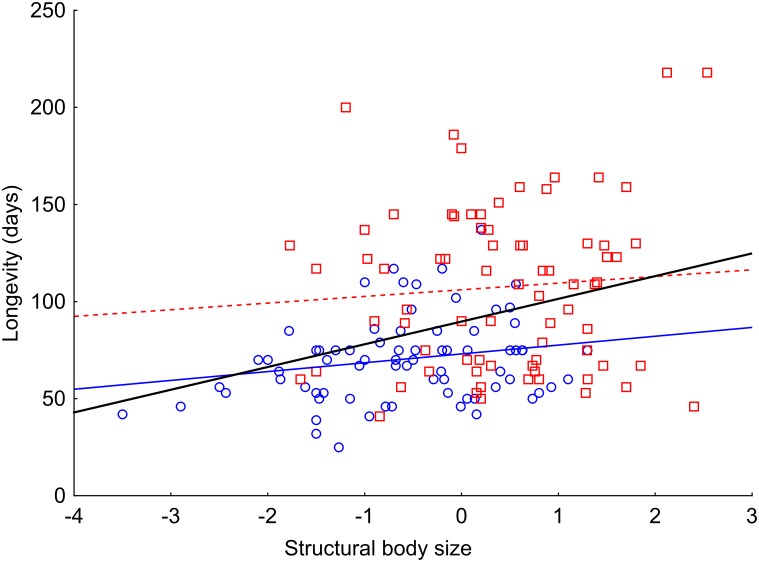
Relationship between starvation resistance and structural body size in *Anchomenus dorsalis*. There seemed to be a significant positive relationship between longevity without food and structural body size when gross effect was tested (data for all beetles pooled = black line). However, the net effects of structural body size became just marginally significant after correction for sex. To visualize this pattern, males are represented by blue circles and females by red squares (sexes were fitted separately). Structural body size is represented by specimen scores from principal component analysis integrating elytron length and pronotum width into a single variable.

Longevity without food was significantly affected by sex, pre-starvation body mass and, to a lesser extent, by structural body size and the interaction between pre-starvation body mass and sex (Table 2). In males, the increase in pre-starvation body mass resulted in a steeper increase of longevity compared to females. In total, these significant variables explained 51.65% of the variation in longevity without food between individuals. The most influential predictor of starvation resistance was pre-starvation body mass (an increase in pre-starvation body mass resulted in prolonged longevity without food). The net effect of pre-starvation body mass, i.e., corrected for structural body size and sex, accounted for 21.58% of the total variation in beetle longevity (Fig 3A). The net effect of sex explained 8.14% of the total variation (females survived for a longer period without food than males; Fig 3B). The most resistant female survived for 218 days without food, and the most resistant male survived for 137 days. Interestingly, the positive net effect of structural body size (corrected for sex) was only marginally significant and explained only 1.50% of the total variation (Table 2; Fig 4). For results obtained using the Cox proportional hazards model, see S1 Table.

**Table 2:** Factors affecting starvation resistance in *Anchomenus dorsalis*. The reported net effects of structural body size, sex and pre-starvation body mass are based on the variance partitioning technique applied to a generalized linear model with a quasi-Poisson distribution of errors. Significant effects are highlighted in **bold**.

Term	Df	Deviance	F-value	P-value	Variation explained (%)
Structural body size^#^	1	34.52	4.41	0.038	1.50
Sex^#^	1	187.35	24.13	<0.001	8.14
Pre-starvation body mass^#^	1	496.58	63.37	<0.001	21.58
Sex * Pre-starvation body mass	1	59.80	7.70	0.006	2.60
Total explained deviance^$^		1188.50			51.65
Residual deviance	143	1112.60			

^#^ Net effect values are shown

^$^ Including variation (deviance) shared among particular terms

### Fat content and lean dry mass changes

**Fig 5:**
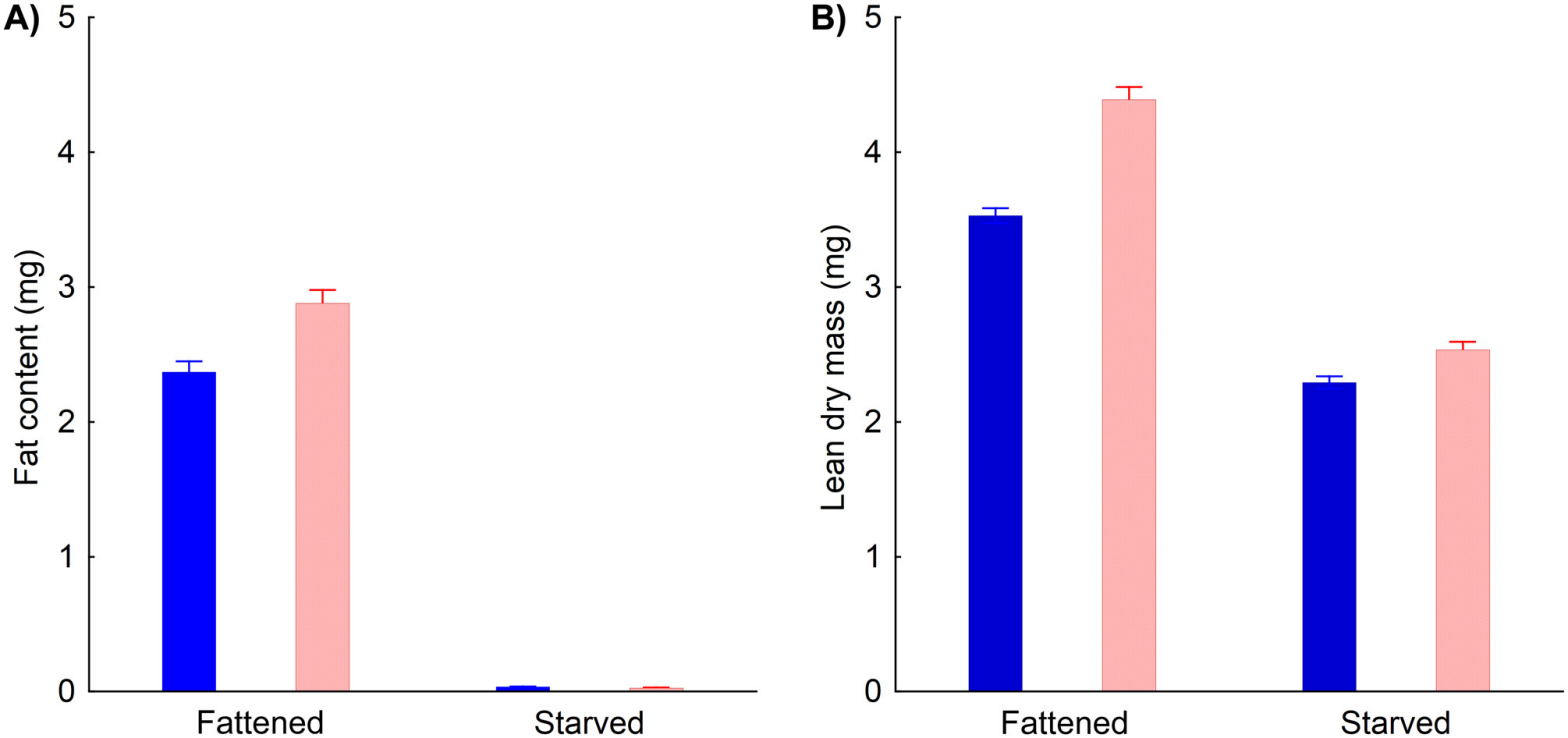
Fat content and lean dry mass in fattened and starved *Anchomenus dorsalis*. Absolute A) fat content and B) lean dry mass are reported separately for males (blue columns) and females (pink columns), both fattened and starved. Means ± SE are shown.

Fat stores were almost completely exhausted during the starvation period (Fig 5A), and there was also a significant interaction between starvation and sex. Fattened females had higher fat content than fattened males, but after starvation there was a minimal difference between sexes (Fig 5A). Absolute fat content was also affected by pre-starvation body mass. Relatively heavier beetles, i.e., these in better body condition, have higher absolute fat content than beetles with relatively lower pre-starvation body mass (Table 3).

**Table 3:** Effects of starvation, structural body size, sex and pre-starvation body mass on the fat content and lean dry mass of *Anchomenus dorsalis*. The reported net effects of starvation, structural body size, sex and pre-starvation body mass on absolute fat content or absolute lean dry mass are based on the variance partitioning technique applied to a generalized linear model with a gamma distribution of errors and a log link function. A dataset including 100 beetles fattened ad libitum and 99 starved beetles was employed in analyses of absolute values. The reported net effects of structural body size, sex and pre-starvation body mass on relative fat content or relative lean dry mass are based on the variance partitioning technique applied to a generalized linear model with a gamma distribution of errors and an inverse link function. A dataset including 100 beetles fattened ad libitum was employed in the analyses of relative values. Significant effects are highlighted in **bold**. Non-significant main effects were included in the final models as the covariates necessary to compute net effects for other terms.

Response	Term	Df	Deviance	F-value	P-value
Absolute fat content	Starvation treatment^#^	1	589.46	1400.10	**<0.001**
	Structural body size^#^	1	0.11	0.27	0.605
	Sex^#^	1	1.18	2.91	0.089
	Pre-starvation body mass^#^	1	2.39	5.90	**0.016**
	Sex*Starvation treatment	1	1.84	4.38	**0.038**
	Total explained deviance^$^		610.09		
	Residual deviance	193	73.34		
Absolute lean dry mass	Starvation treatment^#^	1	10.742	3700.60	**<0.001**
	Structural body size^#^	1	0.526	181.04	**<0.001**
	Sex^#^	1	0.011	3.61	0.059
	Pre-starvation body mass^#^	1	0.172	59.15	**<0.001**
	Pre-starvation body mass*Starvation treatment	1	0.094	32.39	**<0.001**
	Structural body size*Starvation treatment	1	0.073	24.78	**<0.001**
	Sex*Starvation treatment	1	0.195	66.16	**<0.001**
	Total explained deviance^$^		16.860		
	Residual deviance	191	0.562		
Relative fat content	Structural body size^#^	1	0.059	2.17	0.144
	Sex^#^	1	0.033	1.35	0.248
	Pre-starvation body mass^#^	1	0.258	10.60	**0.002**
	Total explained deviance^$^		0.350		
	Residual deviance	96	2.505		
Relative lean dry mass	Structural body size^#^	1	0.000	0.38	0.847
	Sex^#^	1	0.078	30.34	**<0.001**
	Pre-starvation body mass^#^	1	0.134	51.85	**<0.001**
	Total explained deviance^$^		0.212		
	Residual deviance	96	0.250		

^#^ Net effect values are shown

^$^ Including variation (deviance) shared among particular terms

**Fig 6:**
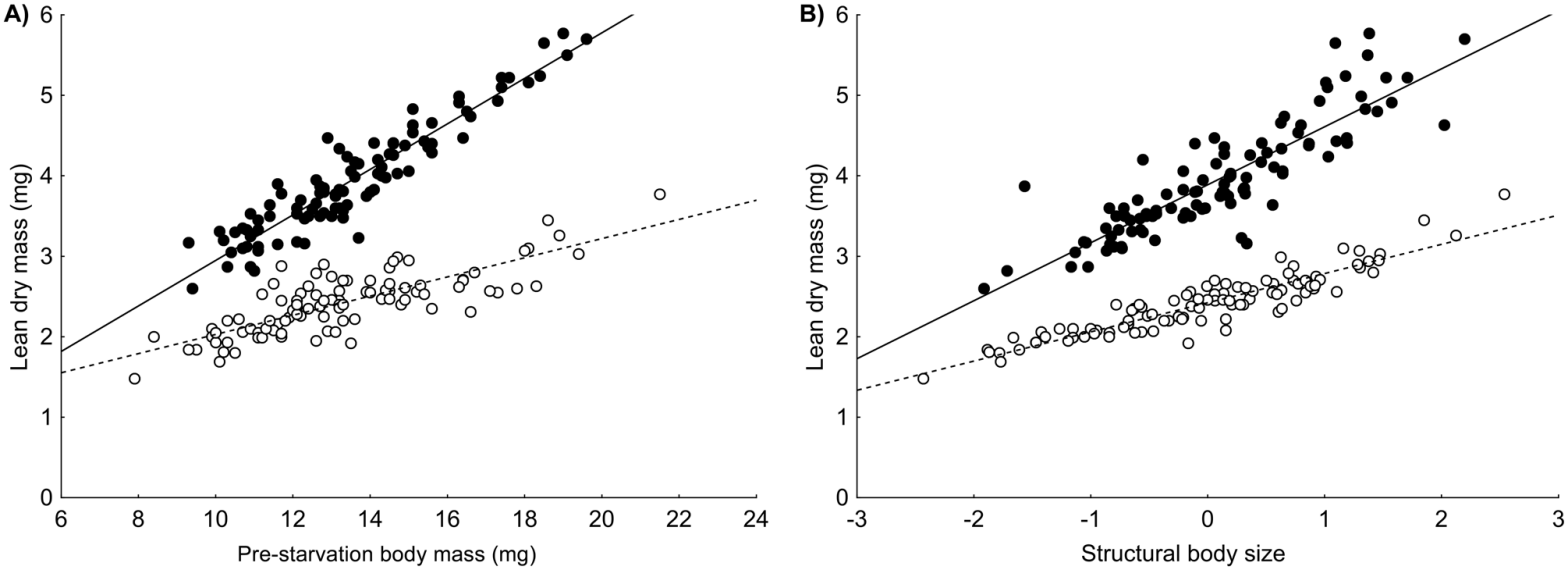
Effects of pre-starvation body mass and structural body size on lean dry mass of *Anchomenus dorsalis*. A) The relationship between pre-starvation body mass and lean dry mass of fattened (full circles) or starved (open circles) beetles. B) The relationship between structural body size and lean dry mass of fattened (full circles) or starved (open circles) beetles. Both panels show significant interactions (A) between starvation treatment (fattened or starved beetles) and pre-starvation body mass, B) between starvation treatment and structural body size.

The absolute lean dry mass of the experimental beetles was significantly reduced during the starvation period (Fig 5B). Lean dry mass was higher in individuals with higher pre-starvation body mass, and this pattern was driven mainly by fattened individuals (Fig 6A). Absolute lean dry mass increased with increasing structural body size, and this relationship was especially obvious for fattened beetles (Fig 6B). The net effect of sex (after correction for the effects of structural size, pre-starvation body mass and starvation treatment) was marginally insignificant; however, there was a significant interaction between sex and starvation treatment. Lean dry mass was reduced more substantially during starvation in females than in males (Fig 5B). Detailed results of the statistical analyses are shown in Table 3.

The relative fat content of fattened beetles increased significantly with increasing relative pre-starvation body mass and was not affected by sex, structural body size or any interactions (Table 3). The relative lean dry mass of fattened beetles increased significantly with increasing relative pre-starvation body mass and was significantly higher in females than in males (Table 3).

## Discussion

The longevity without food observed in *Anchomenus dorsalis* confirmed the supposed ability of carabid beetles to overcome long periods of starvation, which could be a quite common situation in nature. Two of the *A*. *dorsalis* females in this study survived more than 7 months without food, making this one of the most starvation-resistant species among beetles; its longevity without food approaches that of well-adapted spiders [42]. Compared to other carabid species, *A*. *dorsalis* survived without food for twice as long as *Merizodus soledadinus* and *Calosoma sayi* [9,43]. The increase in post-overwintering body mass resulting from extensive feeding (average male mass increased by 26% and average female mass by 31%) confirms our previous findings that carabid beetles deplete their energy reserves during the overwintering period in temperate climates [32]. The body mass loss values reported for *A*. *dorsalis* in this study are comparable to the values reported for *Merizodus soledadinus* (ca 30% loss) by Laparie et al. [9] and *Calosoma sayi* (ca 27% loss) by Young [43].

The body mass of *A*. *dorsalis* females varied relatively more than males during both post-overwintering ad libitum feeding and during the starvation period. This pattern seems to be quite common among beetles (see, e.g., [43]). There is also evidence that field-collected *A*. *dorsalis* females are able to consume higher amounts of food than males [10]. The females’ ability to acquire higher mass increases could be a cause of their higher starvation resistance. The possible explanation that higher mass variation in *A*. *dorsalis* females is enabled by their higher structural body size is refuted by the absence of a link between relative mass variation and structural body size in this study. This sex-dependent result indicates the existence of some physiological differences between sexes that enables females to increase their mass relatively more than males. The existence of such sex differences is not surprising; sex-specific selection pressure is common in nature due to the different roles of males and females during reproduction. Supposedly, females undergo strong selection to maximize their ability to acquire the additional energy needed for egg production, which will maximize their fitness. However, one must be cautious in interpreting live body mass change consequences for sex-specific starvation resistance because a substantial portion of the total body mass consists of water, and variations in water content can differ between males and females [23]. Some insects are able to compensate for body mass loss due to starvation by increasing their water content [1], and this could obscure the true extent of changes in lipid and protein content during starvation.

The results of this study indicate that body condition before starvation, i.e., pre-starvation body mass, corrected for the effects of structural body size and sex, is a good predictor of starvation resistance in *A*. *dorsalis*. This is in agreement with the results of many studies performed previously on various insect species (e.g., [11,19,44]). The significantly steeper positive relationship between pre-starvation body mass and both fat content and lean dry mass in fattened beetles compared to starved beetles provides evidence that better body condition of *A*. *dorsalis* really corresponds to higher usable energy reserves during starvation. Interestingly, considered by itself, structural body size had only a limited effect on starvation resistance in *A*. *dorsalis* (it explained only 1.5% of the variation in beetle longevity without food). This finding stresses the need to distinguish strictly between structural body size and body mass in studies investigating the ecological and evolutionary consequences of body size.

*Anchomenus dorsalis* females survived on average for more than one month longer without food than males. The difference was not caused by the existence of sexual dimorphism in structural body size (females are significantly larger than males) and only partly by sex-specific body condition (females have higher body mass than males for the same structural body size). The significance of sex after controlling for structural body size and initial body mass thus indicates the existence of some sex-specific physiological traits that enhance longevity without food in females. Possible mechanisms responsible for this pattern are as follows: 1) females contain relatively higher fat and/or protein content, 2) a higher portion of fat and/or protein content is available for energy metabolism in females, i.e., relatively lower residual fat and/or protein content in starved females compared to males, and 3) females use energy more efficiently (inspiration for these mechanisms was taken from Rion and Kawecki [45]). Evidence for sexual dimorphism in traits related to starvation resistance is not scarce in insects. For example *Drosophila leontia* females have higher starvation resistance than males due to the female ability to metabolize glycogen as an energy source [14]. In contrast, *Scathophaga stercoraria* males have been shown to outperform females in starvation resistance due to their larger structural body size, which is linked to higher energy reserves [22]. The results of this study provide evidence for higher relative energy reserves in *A*. *dorsalis* females in the form of resorbable lean dry mass. Fattened females had significantly higher relative lean dry mass than males in this experiment. Moreover, the reduction of absolute lean dry mass due to starvation was much higher in females. The persistence of slightly higher absolute lean dry mass in starved females compared to starved males could be caused by the heavier exoskeleton of larger beetles [46], i.e., females in *A*. *dorsalis*. However, the possibility that *A*. *dorsalis* females simply have a higher efficiency in using their energy store compared to males should be investigated in a future study.

Fat reserves seem to be exhausted relatively quickly in *A*. *dorsalis*. Beetles starved for 3 weeks almost completely depleted their extractable lipids (M. Knapp unpublished data). Thus, long-term survival of *A*. *dorsalis* without food is probably linked to reduction of lean dry mass and reduced activity (metabolism) in starved beetles. No measurements of metabolism intensity were made in this study, but beetle activity decreased substantially after approximately one week of starvation (based on unquantified observations made in the course of the experiment).

In conclusion, this study provides evidence for the exceptional ability of the carabid beetle *Anchomenus dorsalis* to survive for long periods without food. Individual starvation resistance was determined by relative pre-starvation body mass (i.e., body condition), and females had significantly higher starvation resistance than males. This pattern could be linked to higher fat content and especially higher lean dry mass of beetles in better body condition and in females than in males. Interestingly, when considered by itself, structural body size had only a limited effect on starvation resistance in *A*. *dorsalis*, which contradicts the contemporary accepted state of knowledge. Because of widespread sexual size dimorphism in insects, our knowledge of mechanisms generating sexual dimorphism in insect performance under stressful conditions cannot advance without distinguishing between the effects of structural body size and other sex-specific traits.

## Supporting Information

S1 Dataset The raw data in Microsoft Excel’s.xlsx format. (XLSX)

S1 Fig A diagram illustrating the experimental design and crucial results of the study. (JPG)

S1 Table The results of the final Cox proportional hazards model. (XLSX)
