# Supplementary figures and images for: Relative Importance of Sex, Pre-Starvation Body Mass and Structural Body Size in the Determination of Exceptional Starvation Resistance of Anchomenus dorsalis (Coleoptera: Carabidae)
Source: PLoS One. 2016 Mar 15;11(3):e0151459. doi: 10.1371/journal.pone.0151459 (PMC4792388; doi:10.1371/journal.pone.0151459)

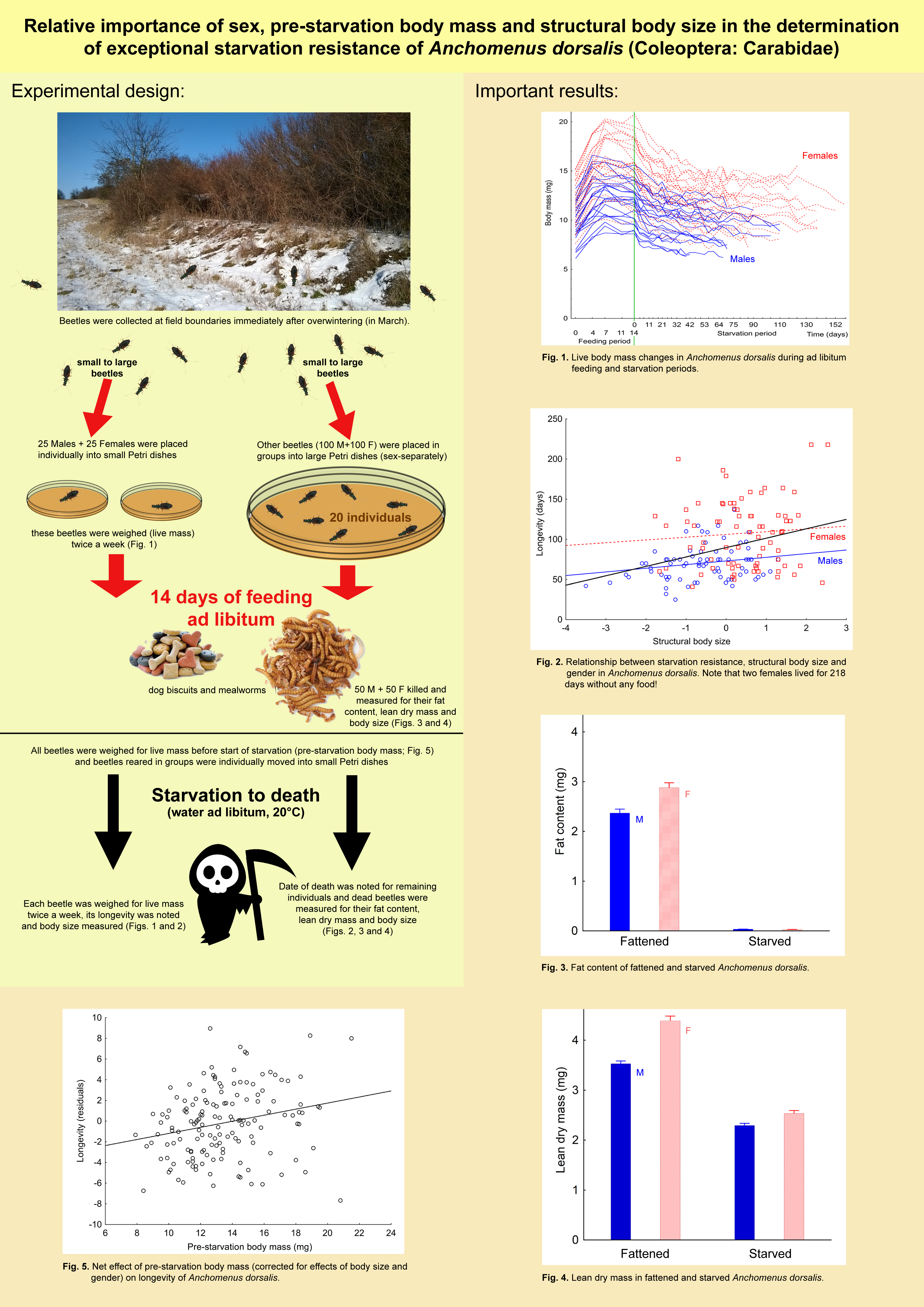

Supplement: S1 Fig — (JPG) [file pone.0151459.s002.jpg]
